# The contribution of the alternative pathway in complement activation on cell surfaces depends on the strength of classical pathway initiation

**DOI:** 10.1002/cti2.1436

**Published:** 2023-01-27

**Authors:** Esther CW de Boer, Astrid JF Thielen, Jeroen D Langereis, Angela Kamp, Mieke C Brouwer, Nienke Oskam, Marlieke L Jongsma, April J Baral, Robbert M Spaapen, Sacha Zeerleder, Gestur Vidarsson, Theo Rispens, Diana Wouters, Richard B Pouw, Ilse Jongerius

**Affiliations:** ^1^ Department of Immunopathology, Sanquin Research and Landsteiner Laboratory Amsterdam Infection and Immunity Institute, Amsterdam University Medical Centre Amsterdam The Netherlands; ^2^ Department of Pediatric Immunology, Rheumatology, and Infectious Diseases, Emma Children's Hospital Amsterdam University Medical Centre Amsterdam The Netherlands; ^3^ Laboratory of Medical Immunology, Radboud Institute for Molecular Life Sciences Radboudumc Nijmegen The Netherlands; ^4^ Radboud Center for Infectious Diseases, Radboudumc Nijmegen The Netherlands; ^5^ Translational and Clinical Research Institute Newcastle upon Tyne UK; ^6^ Department of Hematology, Luzerner Kantonsspital Luzern and University of Bern Bern Switzerland; ^7^ Department for BioMedical Research University of Bern Bern Switzerland; ^8^ Department of Experimental Immunohematology, Sanquin Research, and Landsteiner Laboratory Amsterdam University Medical Center Amsterdam The Netherlands; ^9^ Centre for Infectious Disease Control National Institute for Public Health and the Environment (RIVM) Bilthoven The Netherlands; ^10^ Sanquin Health Solutions Amsterdam The Netherlands

**Keywords:** alternative pathway, amplification loop, antibodies, autoimmune haemolytic anaemia, classical pathway, complement activation

## Abstract

**Objectives:**

The complement system is an important component of innate immunity. The alternative pathway (AP) amplification loop is considered an essential feed forward mechanism for complement activation. However, the role of the AP in classical pathway (CP) activation has only been studied in ELISA settings. Here, we investigated its contribution on physiologically relevant surfaces of human cells and bacterial pathogens and in antibody‐mediated complement activation, including in autoimmune haemolytic anaemia (AIHA) setting with autoantibodies against red blood cells (RBCs).

**Methods:**

We evaluated the contribution of the AP to complement responses initiated through the CP on human RBCs by serum of AIHA patients and recombinant antibodies. Moreover, we studied complement activation on *Neisseria meningitidis* and *Escherichia coli*. The effect of the AP was examined using either AP‐depleted sera or antibodies against factor B and factor D.

**Results:**

We show that the amplification loop is redundant when efficient CP activation takes place. This is independent of the presence of membrane‐bound complement regulators. The role of the AP may become significant when insufficient CP complement activation occurs, but this depends on antibody levels and (sub)class. Our data indicate that therapeutic intervention in the amplification loop will most likely not be effective to treat antibody‐mediated diseases.

**Conclusion:**

The AP can be bypassed through efficient CP activation. The AP amplification loop has a role in complement activation during conditions of modest activation via the CP, when it can allow for efficient complement‐mediated killing.

## Graphical abstract

The alternative pathway (AP) amplification loop is considered an essential feed forward mechanism for activation of the complement system. On physiologically relevant surfaces of red blood cells and bacteria, we show that the AP can be bypassed if classical pathway (CP) activation is strong, due to high antibody levels or antibody class. The AP has a role in complement activation during low activation via the CP, when it can allow for efficient complement‐mediated killing.

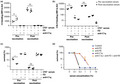

## Introduction

The complement system is part of innate immunity and consists of approximately 50 circulating and membrane‐bound proteins. The system is essential for clearance of pathogens, immune complexes and cellular debris from the human body. Moreover, the complement system is important for homeostasis and has intracellular functions.^1^, ^2^, ^3^ Despite its importance in the innate immune system, it is well‐recognised that the potent inflammatory effects of complement activation can be destructive for host cells. To prevent this, the system is tightly regulated by complement regulatory proteins.^4^, ^5^ The importance of these regulators is shown by the fact that mutations in complement regulators are linked to a large variety of complement‐mediated diseases.^6^, ^7^, ^8^, ^9^ Furthermore, hyperactivation of the complement system by autoantibodies may result in autoimmune diseases such as autoimmune haemolytic anaemia (AIHA), which is characterised by autoantibodies directed against red blood cells (RBCs) that activate the complement system, causing clearance of RBCs through extravascular or intravascular haemolysis.^10^, ^11^

The complement system can be activated via three pathways: the classical (CP), the lectin (LP) or the alternative pathway (AP) dependent on the nature of the activating ligand. The CP is activated by antibody–antigen complexes, the LP by pathogen‐specific carbohydrates and the AP is either activated spontaneously or by C3b as downstream amplifier of the CP or LP. During activation of the CP and the LP, complement proteins C4 and C2 are cleaved, forming the C3 convertase C4b2b on surfaces. The spontaneous activation of the AP is activated by the natural hydrolysis of C3 into C3(H_2_O), allowing for the binding of factor B (FB), which in turn is cleaved by the serine protease factor D (FD) resulting in the C3 convertase C3(H_2_O)Bb. Furthermore, the AP can be activated by C3b formed by the CP or LP, also known as the amplification loop.^1^, ^12^ After the formation of C3 convertases, C3 cleavage induces C5 convertase formation and terminal complement activation.^1^

Complement activation drives several diseases, and although the main complement route at play is often clear, the role of additional pathways is not always fully understood. Some pathologies, such as age‐related macular degeneration, atypical haemolytic uremic syndrome and C3 glomerulopathy, are thought to depend on AP activation.^7^, ^13^, ^14^, ^15^ This is demonstrated by the fact that these diseases are linked to mutations in (regulators of) the AP.^16^, ^17^, ^18^, ^19^ For other, mainly antibody‐driven diseases such as AIHA and rheumatoid arthritis (RA), CP activation is key, but the role of the LP and AP in disease development and progression is often unclear.^11^, ^20^, ^21^ This knowledge is highly relevant when developing disease‐specific complement‐targeting drugs.^6^ The two complement therapeutics currently available in the clinic block C3 and C5^22^, ^23^ and thus do not target a specific activation pathway. However, complement therapeutics targeting specific complement activation pathways are emerging, such as the recently approved anti‐C1s sutimlimab,^24^ for which a better understanding of the role of the AP in different diseases and contexts will be important.^6^, ^11^, ^25^, ^26^

Based on experiments using artificial surfaces devoid of complement regulators, it has been postulated that the AP has a large contribution to total complement activation (up to 80%) through amplification of the response after initial CP or LP activation.^27^, ^28^ The role of the AP‐mediated amplification during complement activation on physiologically relevant cell surfaces is poorly described, but previous evidence points towards a far smaller contribution during antibody‐mediated complement‐dependent cytotoxicity on cancer cells.^29^ As several novel therapeutics are in development that aim to block the AP,^11^, ^30^, ^31^, ^32^ it is crucial to generate fundamental understanding of the role of AP amplification after CP and LP activation on physiological relevant surfaces. This may help to determine whether blocking the AP in certain diseases is beneficial and to which extent complement activation via the other routes remains intact upon AP inhibition. Here, we aimed at getting a further understanding of the conditions that influence the role of the amplification loop after CP activation. We evaluate its contribution to complement activation on human cells upon induction by autoantibodies from AIHA serum or recombinant antibodies, as well as to complement‐mediated bacterial killing. We show that the role of the AP‐mediated amplification after CP‐mediated complement activation depends on the strength of the initial CP activation, since the effect of AP amplification is mainly evident at low antibody concentrations. The amplification via the AP can become redundant at efficient CP activation by high antibody concentrations, leaving the CP‐mediated opsonisation and lysis intact upon AP inhibition. Thus, inhibition of the AP could be beneficial in diseases where the AP is shown to be involved, but our data indicate that AP inhibition may not be beneficial in antibody‐mediated disease.

## Results

### Complement deposition on human cells in AIHA is solely CP‐mediated

**Figure 1:**
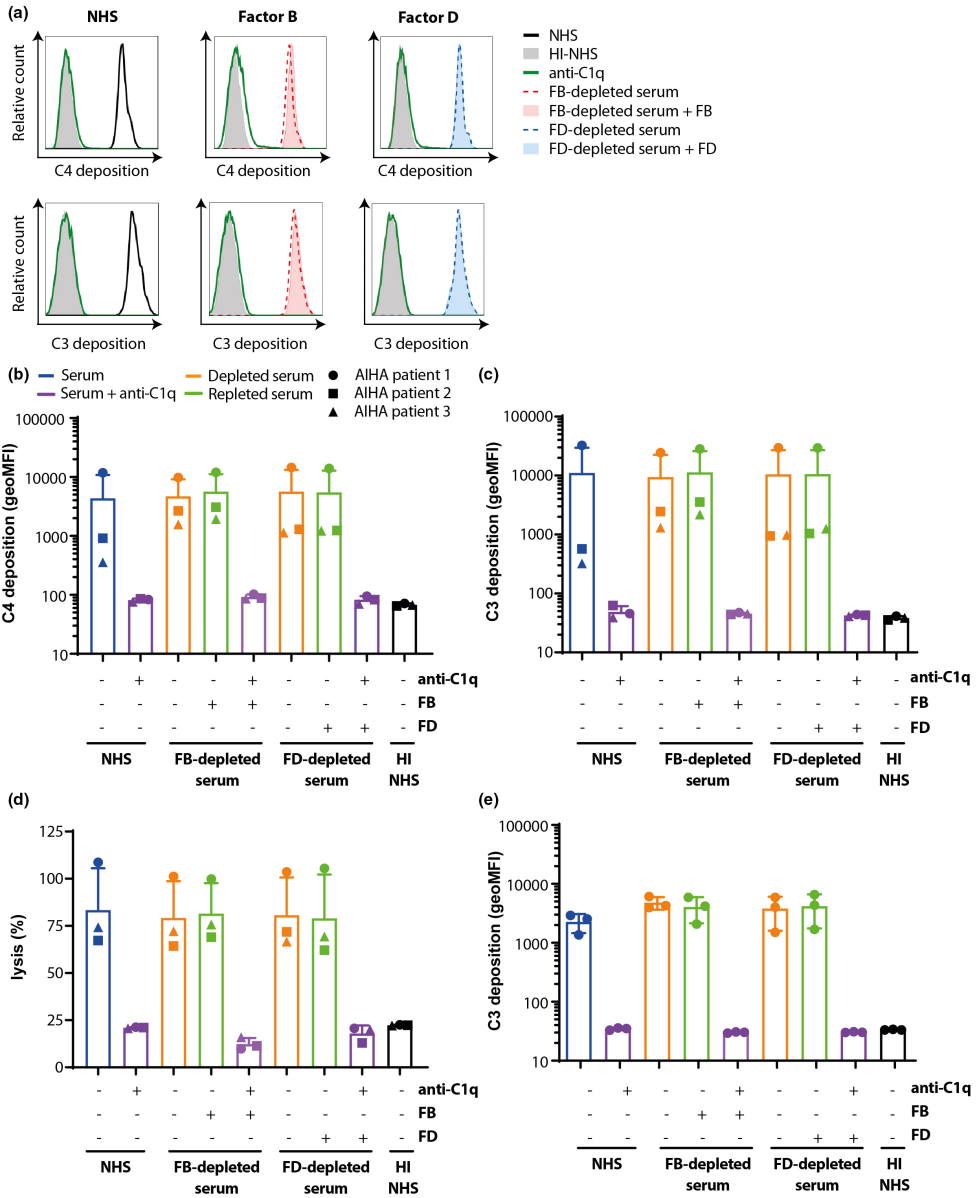
Complement activation in AIHA is fully CP‐mediated. **(a)** Sera of AIHA patients and NHS as complement source (25%, v/v) were incubated with healthy donor RBCs in the presence or absence of CP inhibitor anti‐C1q. C4 and C3 deposition was determined (black line) showing that incubation with anti‐C1q (green line) completely inhibited complement activation to background levels (HI‐NHS solid grey). **(b, c)** Sera of three AIHA patients (with ●, ▲ and ■ depicting individual patients) and FB‐depleted serum (red line) or FD‐depleted serum (blue line) supplemented with or without FB and FD respectively (solid blue/red versus dotted line) as complement source (25%, v/v) were incubated with healthy donor RBCs in the presence or absence of CP inhibitor anti‐C1q (green line) and C4 **(a, b)** or C3 **(a–c)** deposition was determined. C4 and C3 deposition in the absence or presence of FB or FD was equal in all situations. Anti‐C1q completely inhibited complement activation under all conditions. **(d)** Haemolysis of RBCs induced by AIHA patient antibodies, relative to 100% lysis by incubation with milliQ. **(e)** C3 deposition was measured on HAP1 CD46/CD55 KO cells, showing that in the absence of these complement regulators, the presence of FB or FD did not affect complement activation, while anti‐C1q could completely block complement activation. **(a)** Histograms for AIHA patient 1. **(b–d)** Mean with SD of three different AIHA patient sera. **(e)** Mean with SD of n = 3 independent experiments. Statistical significance was tested using the Friedman test with Dunn's multiple comparisons test for **b, c, e** and using repeated measures ANOVA with Dunnett's multiple comparisons test for **d**.

Previous studies, using artificial surfaces devoid of complement regulators, suggested that the AP contributes majorly to total complement activation after initial CP or LP activation.^27^, ^28^ Here, we set out to determine the effect of the AP during CP activation on human cells. To this end, we used a previously developed assay^33^ in which we mimic the antibody‐mediated disease AIHA by incubating healthy donor RBCs with heat‐inactivated AIHA patient serum as an antibody source, supplemented with normal human serum (NHS) as an exogenous complement source. Complement activation was determined by detecting C4 and C3 deposition on the RBC surface^33^, ^34^ (see Supplementary figure 1a for the gating strategy). C4 and C3 deposition was completely abrogated using a blocking monoclonal anti‐C1q antibody, confirming CP initiated complement activation (Figure 1a). To investigate the contribution of the amplification loop to the total amount of complement activation, FB‐ or FD‐depleted serum was used as complement source, with or without the addition of purified FB or FD, respectively. To verify that FB‐ and FD‐depleted serum were indeed AP inactive, we incubated these sera (with or without the addition of purified FB or FD, respectively) in a Mg‐containing buffer on LPS‐coated 96‐wells plates and measured the amount of C3 deposition via ELISA. No C3 deposition was detected in the depleted sera, while C3 deposition was completely restored to the level of NHS when the depleted sera were supplied with purified FB or FD, respectively (Supplementary figure 2a), confirming that the depleted sera were complement active but devoid of AP activity. C4 (Figure 1a and b) and C3 (Figure 1a and c) deposition levels varied between patients, with highest levels for Patient 1. This was the only patient with IgM autoantibodies, which are known to result in strong complement activation.^11^, ^35^ Strikingly, the levels of both C4 and C3 deposition did not change between serum containing FB or FD or not, suggesting that AP does not play a detectable role in this set‐up. The addition of anti‐C1q resulted in C4 and C3 deposition levels comparable to the negative control, heat‐inactivated NHS (HI‐NHS) (Figure 1a–c), indicating that complement activation in these sera was induced by the CP. Next to C4 and C3 deposition, we determined the haemolysis caused by end‐stage complement activation on RBCs. Incubation of RBCs with AIHA sera mixed with various sera as complement source resulted in comparable levels of complement‐mediated lysis for all three patients, irrespective of whether the additional serum contained FB or FD (Figure 1d). Anti‐C1q inhibited the lysis of RBCs to background levels (HI‐NHS) in all patients (Figure 1d). We observed consistently that lack of the AP did not decrease complement activation, whereas inhibiting the CP reduced it to background level (Figure 1a–d). However, statistical significance for the latter observation was not reached due to high patient heterogeneity and the limited patient numbers.

As these results were not in agreement with previous research in plate set‐ups in which a large role for the AP was found,^27^, ^28^ we hypothesised that this difference might be explained by the presence of membrane‐bound complement regulatory proteins on human cells, which do not exist in an ELISA set‐up.^4^ Therefore, we next investigated the contribution of the amplification loop to complement deposition on human HAP1 cells that lack complement regulators CD46, CD55 and complement receptor 1, which inhibit surface C3 convertases^36^ (see Supplementary figure 1b for the gating strategy). Incubation of these human complement regulator‐deficient cells with NHS resulted in C3 and C4 depositions (Figure 1e and Supplementary figure 3a–d). Similar to our results on RBCs, no difference in C3 and C4 deposition was observed after incubation with FB‐ or FD‐depleted sera with or without purified FB or FD supplementation, and complete inhibition of complement deposition was observed with anti‐C1q (Figure 1e and Supplementary figure 3b, c). This suggested that membrane‐bound complement regulators did not affect the contribution of the AP to total complement activation. When repeating these experiments with factor H (FH)‐depleted serum reconstituted with either FH or FH_62V_, a less potent regulator that allows higher AP activity,^19^ we found no difference between the two variants. Control serum and AP inhibited serum resulted in high levels of C4 and C3 deposition for both FH variants, while inhibiting the CP strongly reduced complement deposition (Supplementary figure 3e, f). Thus, even with higher AP activity, no role for AP amplification following CP activation was observed. Altogether, these data demonstrated that *in vitro* complement activation on human RBCs by antibodies from AIHA serum, but also on HAP1 cells lacking membrane‐bound complement regulators, was predominantly mediated by the CP with no detectable contribution of the AP.

### The contribution of the AP in CP‐mediated complement activation is determined by antibody density and class

**Figure 2:**
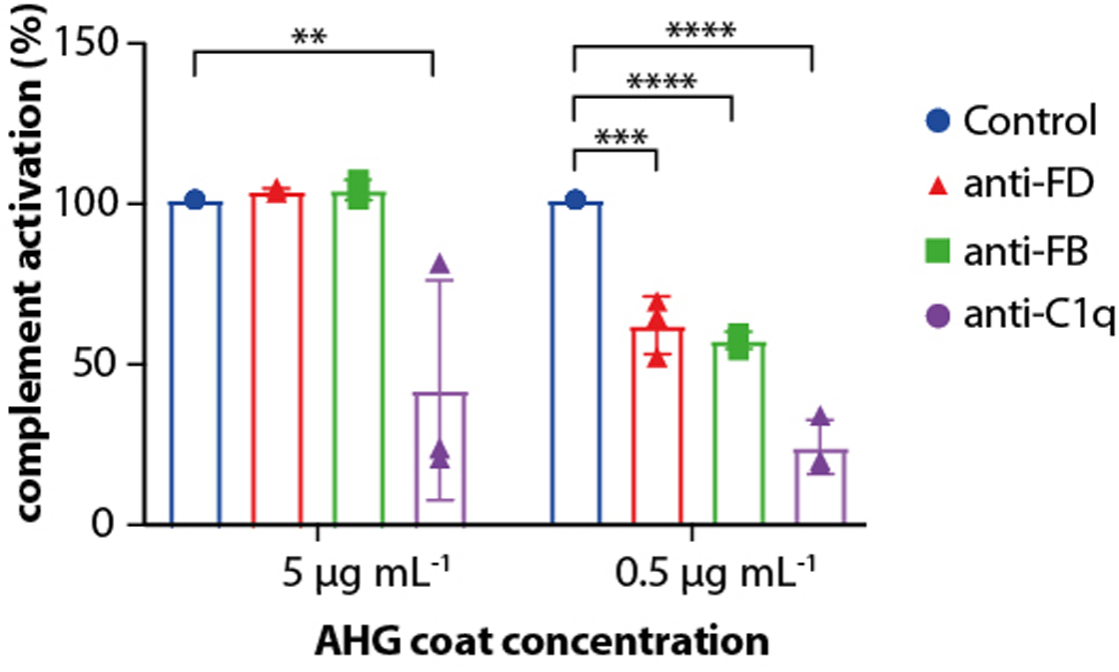
Contribution of the AP to complement activation depends on antibody concentration. In ELISA setting, C3b deposition was measured on varying densities of heat agglutinated IgG (AHG) coat, with 25% (v/v) serum and antibodies to block the alternative or classical pathway, respectively. At high AHG densities, inhibiting the AP with anti‐FB or anti‐FD had no effect on C3b deposition. Bars show mean + SD of *n* = 3 independent experiments, statistical significance was tested using one‐way ANOVA with Dunnett's multiple comparisons test. **P < 0.01, ***P < 0.001, ****P < 0.0001.

As our first results on cells contradicted earlier studies using ELISA set‐ups, we set out to replicate the previous data. These ELISAs were also utilised to further investigate which conditions influence AP contribution and could be followed up upon in cellular assays. As CP activation depends on antibody density,^37^ we varied the antibody concentrations to induce CP activation. To this end, ELISA plates were coated with 10‐fold difference concentrations of heat‐aggregated antibody (AHG), after which 25% human serum was added in the presence or absence of anti‐FB, anti‐FD or anti‐C1q blocking antibodies. The effectiveness of these anti‐FB and anti‐FD antibodies to block the AP was confirmed using an AP‐based ELISA set‐up detecting C3 deposition on coated LPS (Supplementary figure 2b). We observed that with high‐density AHG coat, C3 deposition was not inhibited by anti‐FB and anti‐FD antibodies (Figure 2). Only with low‐density AHG coat, and thus conditions for low CP activation, we observed inhibition at the level of C3b deposition on the ELISA plates when the AP was blocked. Anti‐C1q inhibited complement activation significantly in all conditions. This indicated that the AP contributes to C3 deposition following conditions of low CP activation.

**Figure 3:**
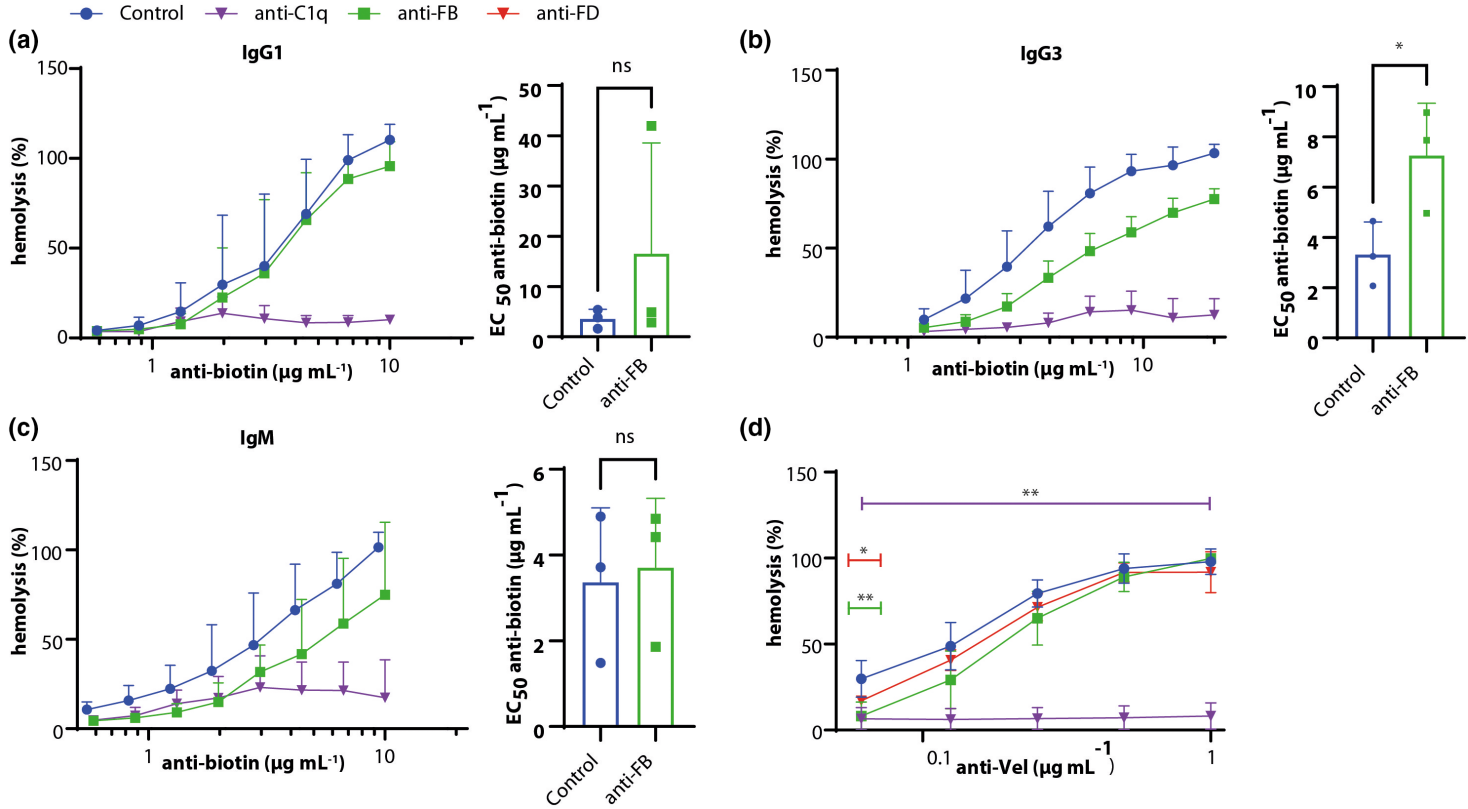
AP contributes to haemolysis upon IgG3‐induced complement activity. Biotinylated RBCs were incubated with IgG1 **(a)**, IgG3 **(b)** or IgM **(c)** antibiotin antibodies, with or without anti‐FB or anti‐C1q to induce haemolysis. The addition of anti‐C1q was able to inhibit haemolysis as induced by IgG1, IgG3 and IgM almost completely. EC50 was calculated to compare control to anti‐FB. With IgG1‐induced activation **(a)**, there was no inhibition by anti‐FB. In IgG3‐induced activation **(b)**, anti‐FB reduced haemolysis at most concentrations and statistically significant increased the EC50. In IgM‐induced activation **(c)**, anti‐FB did not significantly reduce haemolysis or increase the EC50. **(d)** Haemolysis as initiated by an anti‐Vel IgM antibody in a more sensitive system, adding a FH‐inhibiting antibody and bromelain‐treated RBCs. At high anti‐Vel concentrations, there was no effect of inhibiting the AP, but at lowest antibody concentrations, AP inhibition had a statistical significant effect. Blocking the CP inhibited haemolysis completely. **(a–c)** Haemolysis as induced by milliQ was set to 100%. **(a–c)** Mean + SD from *n* = 3, statistical significance tested for EC50 using the paired *t*‐test; **(d)** mean + range from *n* = 2, statistical significance tested using one‐way ANOVA with Dunnett's multiple comparisons test, compared with the serum only condition. * *P* < 0.05, ** *P* < 0.01.

To determine the AP contribution to CP‐initiated complement activation on RBCs, we utilised biotinylated RBCs in combination with monoclonal antibiotin antibodies of different (sub)class.^38^, ^39^ As we used O erythrocytes and AB serum, this system allowed us to control the specific amount and (sub)class of antibody present. We investigated the role of the AP in CP‐mediated haemolysis using human antibiotin IgG1, IgG2, IgG3, IgG4 and IgM. IgG2 and IgG4, which have been described as less potent CP activators in the literature,^40^ did not induce any haemolysis in our system, so the role of the AP could not be determined (data not shown). CP inhibition almost completely reduced haemolysis in all conditions, regardless of antibody (sub)class (Figure 3a–c). Using IgG1 as a complement activation trigger (Figure 3a), the results for control and anti‐FB were similar and the antibiotin EC50 was not affected by AP inhibition. With IgG3, AP inhibition resulted in a curve that is no longer parallel to the control and significantly increased the EC50 of antibiotin (Figure 3b). For IgM, there was no change in parallelism between the anti‐FB and control curve, and the EC50 was unaffected (Figure 3c). IgM is a highly potent CP activator that does not require oligomerisation before C1q binding, unlike IgGs,^35^, ^37^ which could explain the lack of AP contribution at all tested IgM concentrations.

In order to further understand whether the AP can play a role upon IgM‐triggered CP activation, we used an additionally sensitised haemolytic assay, utilising bromelain‐treated RBCs and a FH‐inhibiting antibody combined with anti‐Vel IgM antibodies to induce complement activation. Anti‐C1q inhibited the anti‐Vel‐mediated complement activation completely showing a significant reduction in all anti‐Vel concentrations, clearly indicating that lysis of the RBCs in this system was predominantly CP‐mediated. AP inhibition only decreased the complement‐mediated lysis of RBCs at low anti‐Vel concentrations (Figure 3d), with a significant reduction only at 0.06125 μg mL^−1^ anti‐Vel. In agreement with our ELISA data and the antibiotin IgM data, this indicated that the AP is an important contributor to the CP when the initial activation, due to lower antibody titers, is less robust, even when the AP is dysregulated by FH inhibition.

Overall, using ELISA and cell‐based assays, we show that the contribution of the AP on CP activation is only observed when limited antibody levels, or less potent activators, spark the initial CP‐mediated complement activation.

### Classical pathway complement activation on bacterial surfaces bypasses the amplification loop in the presence of antibodies

**Figure 4:**
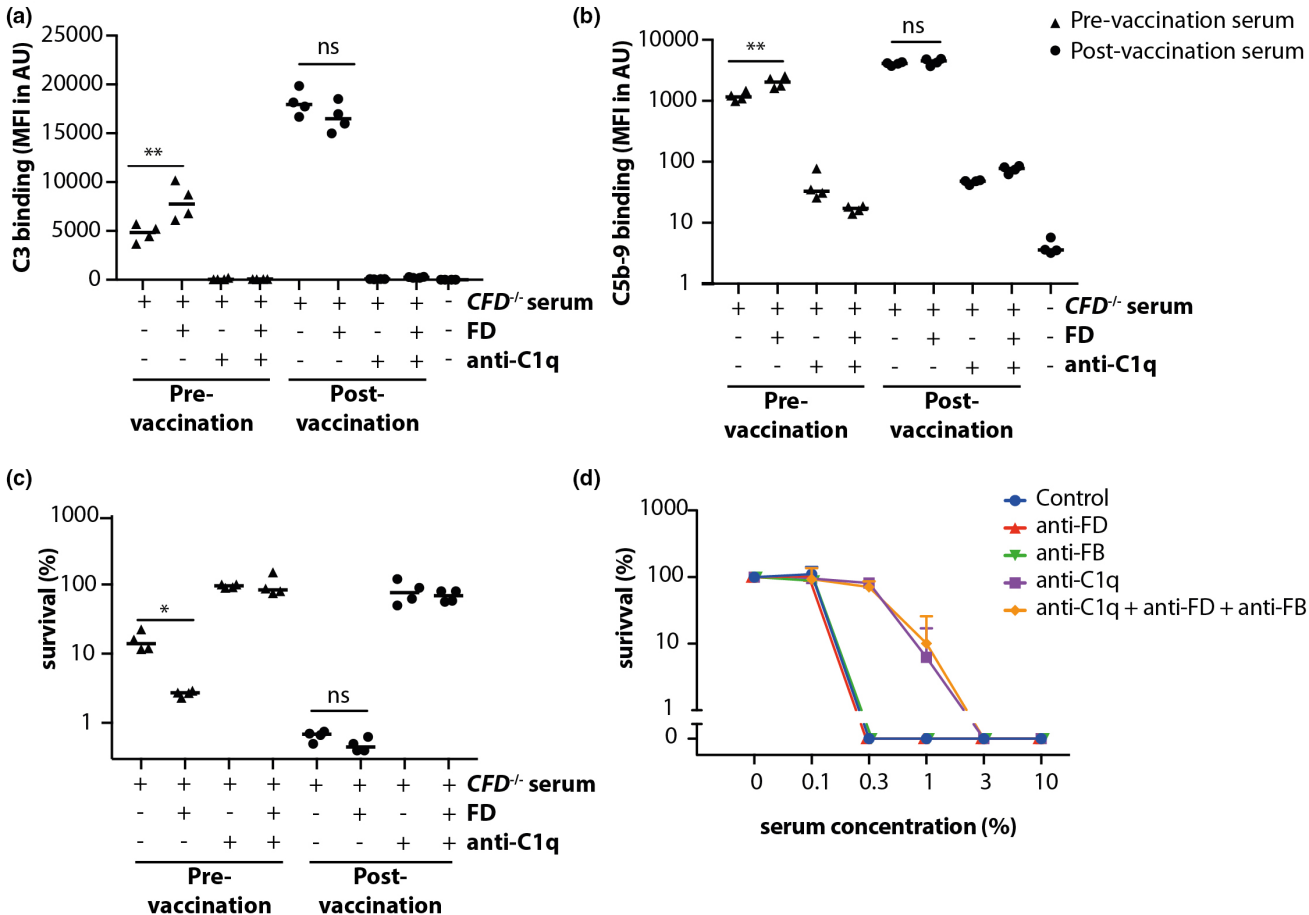
Complement activation on *Neisseria meningitidis* bypasses the amplification loop in post‐vaccination serum. *Neisseria meningitidis* was incubated with 10% FD‐deficient patient serum before (▲) and after (●) MenB‐4C vaccination. **(a, b)** show C3 and C5b‐9 binding, respectively, as determined by flow cytometry on whole bacteria. Both are statistically significant increased by the addition of FD to the deficient serum before vaccination, but this effect was lost postvaccination. The addition of anti‐C1q resulted in complete inhibition of C3 deposition and strong reduction of C5b‐9 deposition in all circumstances. **(c)** Bacterial survival upon serum incubation as determined by CFU, demonstrating that bacterially killing was increased by addition of FD in the prevaccination serum, but not the postvaccination serum. When anti‐C1q was added, bacterial survival was around 100%, indicating that CP activation was required for bacterial killing. **(d)** Complement‐mediated *E. coli* killing was inhibited by addition of anti‐C1q alone to a similar extent as addition of anti‐C1q, anti‐FB and anti‐FD combined. Anti‐FB and anti‐FD alone were unable to prevent complement‐mediated bacterial killing. **(a–c)** Mean and individual data points from four independent experiments, **(d)** mean + SD from *n* = 3 independent experiments. Statistical significance in **a–c** was tested using the paired *t*‐test, * *P* < 0.05, ** *P* < 0.01.

The complement system is vital to ward off invading pathogens, such as bacteria. To take a broader look at the contribution of the AP to CP complement activation on biological surfaces, we investigated the role of the amplification loop during opsonisation and lysis of the bacterial pathogen *Neisseria meningitidis*. To this end, we used serum of a genetically FD‐deficient patient before and after vaccination against *N. meningitidis*.^41^, ^42^ A previous study reported antibodies reactive with *N. meningitidis* in this patient prevaccination, and increased antibody titre after vaccination.^42^ C3 deposition (Figure 4a), C5b‐9 deposition (Figure 4b) in FACS (see Supplementary figure 1c for gating strategy) and killing (Figure 4c) of *N. meningitidis* by FD‐deficient serum was low and could be significantly increased by addition of FD in prevaccination serum. This difference by FD addition was lost in serum of the same patient after vaccination against *N. meningitidis*. Anti‐C1q completely blocked complement activation and bacterial killing in all conditions (Figure 4a–c). This indicates that the AP only plays a measurable role during complement opsonisation and killing of *N. meningitidis* in the absence of a robust antibody response, similar as observed for RBCs and HAP1 cells (Figure 2d and e and Figure 3).

As *N. meningitidis* has specific complement evasion mechanisms, such as FH binding to inhibit AP activation, results observed with *N. meningitidis* might not be applicable to other bacteria. To explore whether these findings are consistent with other bacteria, we also studied the role of the AP using *Escherichia coli* as a model organism. Bacterial killing did not occur upon incubation with HI‐NHS (data not shown) or NHS with eculizumab (Supplementary figure 4), indicating that bacterial killing was terminal complement‐mediated. Upon the addition of anti‐C1q to serum, we observed strong inhibition of bacterial killing. No additional effect was observed when combining anti‐C1q, anti‐FB and anti‐FD (Figure 4d). Individually, the addition of anti‐FB or anti‐FD to NHS showed no inhibition of complement‐mediated killing.

In conclusion, the role of the AP on bacterial surfaces *in vitro* was similar to our observations with human RBCs and seems restricted to situations in which limiting amounts of antibodies are present.

## Discussion

Here, we studied the role of the AP during CP‐initiated complement activation on physiologically relevant human cell surfaces and bacteria. In contrast to what was reported previously in ELISA‐based assays,^28^ the role of the AP on cell surfaces is not as prominent and is determined by the robustness of the initial antibody trigger of the CP.

Understanding the role of the AP in disease is important as a large variety of specific AP blocking therapeutics are currently in development.^11^, ^30^, ^31^, ^32^ To date, the role of the AP in CP‐ and LP‐mediated complement activation is considered to be extensive based on ELISA data detecting terminal complement activation.^27^, ^28^ However, ELISA plates are substantially different from human cells, as they are devoid of membrane‐bound complement regulators which are known to be of great importance during several complement‐mediated diseases.^5^, ^6^ We show that the AP plays a significant role in ELISA at low antibody levels, where the residual complement activation upon anti‐C1q addition could be explained by spontaneous AP activation on the plastic plate or by LP activation. We then assessed the role of the AP in CP‐mediated complement activation on human and bacterial cell surfaces. We found that the role of the AP in CP‐mediated complement activation on surfaces is limited and restricted to situations where only modest levels of antibodies are present, resulting in less robust complement activation. AP contribution was not affected by membrane‐bound complement regulators. This even holds when the AP has become dysregulated by the presence of less potent FH. Thus, the AP‐mediated amplification seems mainly relevant when complement activation by the CP is modest. This means that the AP becomes redundant when antibodies can activate the CP strongly because of a high titre, or because of strong C1q binding as is seen for IgM.^43^ This observation is in agreement with earlier studies where the role of the AP was nearly undetectable when efficient CP activation was induced by HexaBodies^29^ and *in vitro* blocking of the AP did not affect complement activation on *Streptococcus pneumoniae* and *N. meningitidis* in vaccinated serum.^44^, ^45^

Previous research has mainly used murine models to assess the contribution of the AP in CP‐mediated diseases. Models for RA, antiphospholipid syndrome and myasthenia gravis indicated involvement of AP activation in disease outcome.^46^, ^47^, ^48^, ^49^ This seems contradictory to data presented in this paper. However, several details should be taken into account to put our data in perspective with previous animal research. First, the murine models for complement‐mediated diseases cannot directly be translated to human conditions as complement activation varies between mouse strains and even the gender of the mice seems to have influence on complement activity.^50^ Mice also have different complement regulatory proteins than humans, which makes it difficult to extrapolate results from murine models to the human situation.^50^, ^51^ For example, mice only have three instead of the human five FH related‐proteins (FHRs),^52^ a different expression pattern of most membrane‐bound complement regulators and mice express Crry instead of CR1.^51^ Second, our results indicate differences between antibody (sub)classes and titres, especially between IgG and IgM, which other studies did not compare (sub)classes. Lastly, when looking into RA specifically, inflammation and complement activation occurs in the joints and on cartilage, as well as systemically. Although it is generally accepted that the CP is strongly involved in RA via both IgG and IgM antibodies in the joints,^53^ previous clinical research showed systemic involvement of both the AP and the CP.^54^ It is suggested that immune complexes on the articular surface initiate an inflammatory cascade via the AP in mice.^46^, ^55^ The mice model in these papers mainly depends on IgG1 antibodies, which are known to be a poor activator of the murine CP.^46^ This could explain the observation of the strong involvement of the AP here: the amplification loop of the AP kicks into play when CP activation is low as we have shown in Figures 3 and 4.

Relevance of the AP in the killing of bacteria has been shown *in vivo* in some studies, particularly mouse models. Many pathogenic bacteria use FH binding as complement evasion strategy,^56^, ^57^ indicating the importance of the AP *in vivo*. One patient with FB deficiency was reported to suffer from pneumococcal infections and meningitis caused by *N. meningitidis*,^58^ which seized upon vaccination and antibiotic prophylaxis. Furthermore, genetic FD deficiency has been described to be associated with meningococcal infections in humans.^41^, ^59^, ^60^ These studies do not report on initial antibody titers against meningococci, and most cases describe young, unvaccinated infants. With regard to animal studies, C3 opsonisation of *S. pneumoniae* was lower when serum of FD‐deficient mice was used, than serum of FD‐sufficient mice.^61^ In line with these results, C3 opsonisation of *S. pneumoniae* in FB‐deficient mice was reduced compared to wild‐type mice, in addition to lower phagocytic capacity.^62^ Discrepancies between these previously published papers and our experiments on bacterial surfaces regarding the role of the AP might be explained by the fact that laboratory mice most likely do not have specific antibodies, and therefore have minimal CP activation, against the described bacterial pathogens. This is in agreement with our current data (Figure 4a–c) and previous publications that show that the role of the AP diminishes upon vaccination.^44^, ^45^

Taken together, lacking a functional AP by missing FD or FB seems to result in higher susceptibility for certain bacteria in both mice and humans. Based on our current results, we conclude that the AP is important for the clearance of pathogens by C3b opsonisation and membrane attack complex formation upon first encounter with the pathogen, when there is no efficient antibody response yet. When antibodies have been formed through exposure or vaccination, the CP may be activated via these antibodies in which case the AP amplification loop is not required, as underlined by our current data. Future research could investigate how the combination of different antibody (sub)classes within a polyclonal antibody response would influence the contribution of the different activation pathways. One interesting follow‐up question would then be to investigate whether the presence of IgG2 or IgG4 antibodies, which do not (strongly) activate complement, would lead to competition with complement‐activating (sub)classes for epitope binding, or reduce hexamerisation efficiency, and thus result in less optimal CP activation.

In conclusion, we show that the importance of the AP amplification in CP‐mediated complement activation depends on the robustness of the initial activation trigger. Although we have not studied this for complement activation via the LP, we hypothesise, based on the similarities between the CP and LP complement activation,^1^, ^63^ that this will also apply to the contribution of the AP amplification in LP dependent complement activation. Therefore, intervention in the amplification loop may not be an effective therapy for human CP‐mediated diseases such as AIHA, in which complement activation via antibodies is the sole activation route. Treatment of CP‐initiated autoimmune diseases may be most effective when using complement inhibitory drugs targeting the very initiation of the CP, for example by C1‐esterase inhibitor or sutimlimab,^24^ both inhibiting the CP protease C1s.^64^, ^65^, ^66^, ^67^ Targeting the AP remains relevant in AP‐mediated disease. We have shown that CP activation is not abrogated by AP inhibition if antibody titres are high. This would indicate that the AP can probably be safely inhibited in patients without diminishing CP activity with the additional advice of vaccination against pathogens such as *N. meningitidis*.

## Methods

### Serum material

From a previous study,^68^ three anonymised serum samples of AIHA patients were used as antibody source in which complement was inactivated by incubation for 30 min at 56°C. Patient 1 was diagnosed with IgM class autoantibodies, Patients 2 and 3 with IgG autoantibodies. A NHS pool was created from eight different healthy volunteers after informed consent as used in the C3 and C4 deposition assays on RBCs and HAP1 cells, except for the HAP1 assay using FH_62V_ where we used a pool of 148 healthy volunteers after informed consent. An NHS pool consisting of 30 different healthy volunteers after informed consent was used for the ELISAs and *E. coli* experiments. AB serum was obtained from individual healthy donors after informed consent and used for the haemolytic assays. As a negative control for complement activation, NHS was heat inactivated for 30 min at 56°C (HI‐NHS). FB‐, FD‐ and FH‐depleted serum were obtained from CompTech (Tyler, TX, USA). Serum of a patient with homozygous *CFD* mutation before and after meningococcal ACYW and 4CMenB vaccination was obtained as described by Sprong *et al*.^41^ and van den Broek *et al*.^42^

### Antibodies and proteins

To detect complement deposition in‐house monoclonal antibody (mAb), anti‐C3‐19 (which binds C3 fragments C3b, iC3b, C3d and C3dg) and anti‐C4‐10 (which binds C4b and C4d) were used.^33^, ^69^ In‐house inhibitory mAb anti‐C1q‐85 was used to block the CP^70^ at three times excess of C1q serum concentration, in‐house inhibitory mAb anti‐FB‐1 and anti‐FD (Lampalizumab; Genentech, San Francisco, CA, USA) were used to inhibit the AP at 2.5 times excess of the serum concentration of their respective target. Inhibitory mAb anti‐C5 (Eculizumab; Alexion Pharmaceutical, Cheshire, CT) was obtained from remnants of used Soliris® injection bottles and used to block terminal complement activation to prevent lysis during the experiments. Antibiotin and anti‐Vel antibodies for the haemolytic assays were produced in‐house, using sequences described by Bagçi *et al*.,^39^ Kohen *et al*.,^38^ van der Rijst *et al*.^71^ and Oskam *et al*.^72^ and methods described by Falkenburg *et al*.^73^ In‐house inhibiting mAb anti‐FH.09 was used in the haemolytic assay with anti‐Vel at 2.5 times excess of FH serum concentration.^74^ Purified FB, FD and FH were obtained from CompTech. In all experiments, 75 μg mL^−1^ purified FB and 0.5 μg mL^−1^ purified FD were added to substitute 25% (v/v) FB‐ or FD‐depleted serum, respectively, to approach normal levels of these proteins. FH_62V_ was affinity purified from EDTA plasma.^19^ 300 μg mL^−1^ FH or FH_62V_ was used to reconstitute FH‐depleted serum.

### Alternative pathway and CP C3 deposition ELISA

ELISAs were performed as described by Dekkers *et al*.^75^ For the alternative pathway ELISA, Nunc Polysorp flat‐bottom plates (Thermo Scientific, Rockland, IL, USA) were coated overnight with 40 μg mL^−1^ lipopolysaccharide from *Salmonella typhosa* (LPS, Sigma‐Aldrich, St. Louis, MI, USA) in phosphate‐buffered saline (PBS) at room temperature (RT). Plates were washed and incubated for 1 h at 37°C with 20% (v/v) NHS or FB‐or FD‐depleted serum, with or without purified FB (162.5 μg mL^−1^) or FD (1.05 μg mL^−1^) supplementation in Veronal Buffer (1.8 mm sodium barbital and 3.1 mm barbituric acid, pH 7.3–7.4; VB) with 0.1% (w/v) Tween‐20, 0.3% (w/v) BSA, 5 mm MgCl_2_ and 10 mm EDTA. For the CP ELISA, Nunc Maxisorp flat‐bottom plates (ThermoFisher Scientific, Whaltam, MA, USA) were coated overnight with 5 or 0.5 μg mL^−1^ agglutinated human IgG (AHG, Sanquin Reagents) in PBS at RT. Plates were washed and incubated for 1 h at RT with 25% (v/v) NHS with or without anti‐FB (202 μg mL^−1^), anti‐FD (1.3 μg mL^−1^) or anti‐C1q (31.75 μg mL^−1^) in VB with 0.1% (w/v) Tween‐20, 0.3% (w/v) BSA, 1 mm CaCl_2_ and 0.5 mm MgCl_2_. ELISA was performed as described above. Biotinylated anti‐C3.19 was used as conjugate and ELISA was developed as described by Dekkers *et al*.,^75^ with the exception of the usage of streptavidin‐HRP for the classical pathway ELISA.

### Complement activation on RBCs and human cells deficient for CD46/CD55

C3 and C4 deposition on RBCs was assessed as described previously.^33^ In short, 0.08% (v/v) bromelain‐treated O‐typed RBCs were incubated with 3% (v/v) AIHA patient serum as antibody source, 25% (v/v) serum as complement source (NHS, FB‐ or FD‐depleted serum) and in the presence of at least equimolar inhibitory mAb anti‐C5 in VB with 0.1% (w/v) gelatin (VBG) supplemented with 10 mm CaCl_2_ and 2 mm MgCl_2_ (VBG^++^) for 1.5 h at 37°C. As described previously,^36^ human CD46/55‐deficient HAP1 cells (1.0 × 10^5^ cells) were incubated with 25% (v/v) complement source (NHS, FB‐, FD‐ or FH‐depleted human serum) and at least equimolar inhibitory mAb anti‐C5 in VB^++^ for 1 h at 37°C.DAPI was used at 1 μm to detect dead cells. C3 and C4 deposition was detected by using 1.0 μg mL^−1^ DyLight 488‐conjugated anti‐C3‐19 and 1.0 μg mL^−1^ DyLight 647‐conjugated anti‐C4‐10. LSR Canto II flow cytometer (BD Biosciences, San Jose, CA, USA) was used for measuring and data analysis was performed using the FlowJo software v1.0 (BD Biosciences, San Jose, CA, USA).

### Haemolytic assay induced by AIHA serum

0.4% (v/v) O‐typed RBCs were treated with bromelain (Sanquin Reagents, Amsterdam, NL) opsonised with 10% (v/v) AIHA patient sera were incubated for 1.5 h at 37° C with 25% (v/v) serum as complement source (NHS, FB‐ or FD‐depleted human serum) with or without purified FB or FD supplementation. Lysis was measured as absorbance of the supernatant at 412 nm and corrected for the absorbance at 690 nm with a Synergy 2 plate reader (BioTek Instruments, Winooski, VT, USA) and expressed as percentage of a 100% lysis control (RBCs incubated in MilliQ).

### Haemolytic assay induced by anti‐Vel and antibiotin

To determine the effect of antibody concentration and (sub)class on AP contribution on cells, we used an antibiotin haemolytic assay. O‐typed RBCs were biotinylated in 0.11 mm Pierce Premium Grade Sulfo‐NHS‐LC‐Biotin (Thermo Scientific). Cells were diluted to 1% (v/v) in VBG with 2 mm CaCl_2_ and 1 mm MgCl_2_ and incubated with antibiotin antibodies and 25% (v/v) healthy AB serum, with or without anti‐FB (162.5 μg mL^−1^) and anti‐C1q (75 μg mL^−1^) to inhibit the AP and CP, respectively, at 2–3 times excess. Antibiotin antibodies were added at a maximum concentration of 20 μg mL^−1^ for IgG1 and IgM and at 40 μg mL ^−1^ for IgG2, −3 and − 4 and diluted 1:1.5. Cells were incubated at 37°C for 1 h while shaking. Lysis was measured as absorbance of the supernatant at 412 nm and corrected for the absorbance at 690 nm with a Synergy 2 plate reader (BioTek Instruments) and expressed as percentage of a 100% lysis control (RBCs incubated in MilliQ).

To further assess the role of the AP on RBC surfaces, we used our previously set‐up haemolytic assay that was optimised to detect complement‐activating antibodies by the addition of a FH inhibiting antibody^74^ and using bromelain‐treated RBCs. 25% (v/v) healthy AB serum was pre‐incubated with inhibiting anti‐FH.09 (59.5 μg mL^−1^) and with or without anti‐FD (1.38 μg mL^−1^), anti‐FB (150 μg mL^−1^) and anti‐C1q (75 μg mL^−1^) in VBG with 1 mm CaCl_2_ and 0.5 mm MgCl_2_. Anti‐Vel IgM was added at 1 μg mL^−1^ and titrated 1:2. 0.5% (v/v) bromelain‐treated O‐typed RBCs were added and incubated with serum and antibodies at 37 °C for 1 h while shaking. Lysis was measured as absorbance of the supernatant at 412 nm and corrected for the absorbance at 690 nm with a Synergy 2 plate reader (BioTek Instruments, Winooski, VT, USA) and expressed as percentage of a 100% lysis control (RBCs incubated in MilliQ).

### C3 and C5b‐9 binding to *Neisseria meningitidis*

Bacteria were grown in Tryptic Soy Broth to OD_620_ ~ 0.23, washed once with HBSS + Ca^2+^/Mg^2+^ + 0.1% (w/v) gelatin (HBSS3+) and diluted to an OD_620_ of 0.2 with HBSS3+. Twenty‐five microlitre bacteria was mixed with 25 μL 20% (v/v) patient serum diluted in HBSS3+ with or without reconstitution of 0.2 μg mL^−1^ FD (CompTech) and/or 30 μg mL^−1^ anti‐C1q and incubated 30 min at 37°C. Bacteria were pelleted by centrifugation at 3200× ***g*** and fixed for 20 min in 2% (w/v) paraformaldehyde in PBS at RT. Surface‐bound complement C3 and complement complex C5b‐9 were detected with 1:500‐diluted FITC‐labelled polyclonal goat anti‐human C3 (MP biomedicals) and 1:100‐diluted monoclonal mouse antihuman C5b‐9 (Clone aE11, Santa Cruz Biotechnology, Santa Cruz, CA, USA) followed by 1:500‐diluted Alexa647‐labelled donkey antimouse IgG (Invitrogen). Surface binding of C3 and C5b‐9 was determined by flow cytometry using a FACS LSR II instrument (BD Biosciences, San Jose, CA, USA) and expressed in mean fluorescence intensity (MFI) in arbitrary units (AU). Data were analysed by using FlowJo version 10.4.1 (BD Biosciences).

### *Neisseria meningitidis* killing assay

*Neisseria meningitidis* was grown fresh in GC broth at 37°C and 5% CO_2_ to an OD_620_ of 0.23, washed once with HBSS3+ and diluted 800‐fold in HBSS3+ to obtain a concentration of ~200 000 colony forming unit (CFU) mL^−1^. Twenty microlitre bacteria was mixed with 20 μL 20% (v/v) patient serum or HI‐NHS diluted in HBSS3+ with or without reconstitution of 0.2 μg mL^−1^ FD (CompTech) and/or 30 μg mL^−1^ anti‐C1q blocking antibody and incubated 30 min at 37°C. Samples were diluted 10‐, 100‐, 1000‐fold with PBS and 10 μL droplets were plated on GC agar plates and grown overnight at 37°C and 5% CO_2_. Survival was determined by dividing the CFU counts in patient serum by the CFU count in HI‐NHS after a 30‐min incubation and presented as percentage.

### *Escherichia coli* killing assay

An overnight *E. coli* (MG1655) culture was diluted (1:100) and then grown to an OD_620_ of 0.5 at 37°C. Bacteria were diluted to 10 000 CFU mL^−1^ in sterile RPMI/HAS, and 50 μL of bacteria was incubated with a 1:3 serial dilution of NHS and HI‐NHS, starting at 10% (v/v), with or without anti‐C1q (17 μg mL^−1^), anti‐FD (1.5 μg mL^−1^) and anti‐FB (65 μg mL^−1^) and incubated for 1 h while shaking at 37°C. Bacteria were plated at four serial dilutions of 1:10 in PBS on agar plates and incubated overnight at 37°C. CFU were counted the next day.

### Statistics

Analysis and statistical tests were performed using GraphPad Prism (version 9.1.1; GraphPad Software, San Diego, CA, USA).

## Author contributions

**Esther CW de Boer:** Conceptualization; data curation; formal analysis; investigation; methodology; visualization; writing – original draft; writing – review and editing. **Astrid JF Thielen:** Conceptualization; data curation; investigation; methodology; writing – original draft; writing – review and editing. **Jeroen D Langereis:** Data curation; formal analysis; investigation; writing – review and editing. **Angela Kamp:** Investigation. **Mieke C Brouwer:** Investigation. **Nienke Oskam:** Methodology; writing – review and editing. **Marlieke LM Jongsma:** Methodology. **April J Baral:** Methodology; writing – review and editing. **Robbert M Spaapen:** Methodology; writing – review and editing. **Sacha Zeerleder:** Conceptualization; methodology. **Gestur Vidarsson:** Methodology; writing – review and editing. **Theo Rispens:** Methodology; writing – review and editing. **Diana Wouters:** Conceptualization; methodology; writing – review and editing. **Richard B Pouw:** Methodology; supervision; writing – review and editing. **Ilse Jongerius:** Conceptualization; funding acquisition; methodology; supervision; writing – original draft; writing – review and editing.

## Conflict of interest

MCB, TR, DW, RBP and IJ are co‐inventors of (multiple) patents and/or patent applications describing the therapeutic use of anti‐FH antibodies. All other authors declare that the research was conducted in the absence of any commercial or financial relationships that could be construed as potential conflict of interest.

## Supporting information


Supplementary figures 1–4
