# Supplementary material for: The contribution of the alternative pathway in complement activation on cell surfaces depends on the strength of classical pathway initiation
Source: Clin Transl Immunology. 2023 Jan 27;12(1):e1436. doi: 10.1002/cti2.1436 (PMC9881211; doi:10.1002/cti2.1436)
Supplement: Supplementary file 1 — Supplementary figures 1–4 [file CTI2-12-e1436-s001.pdf]

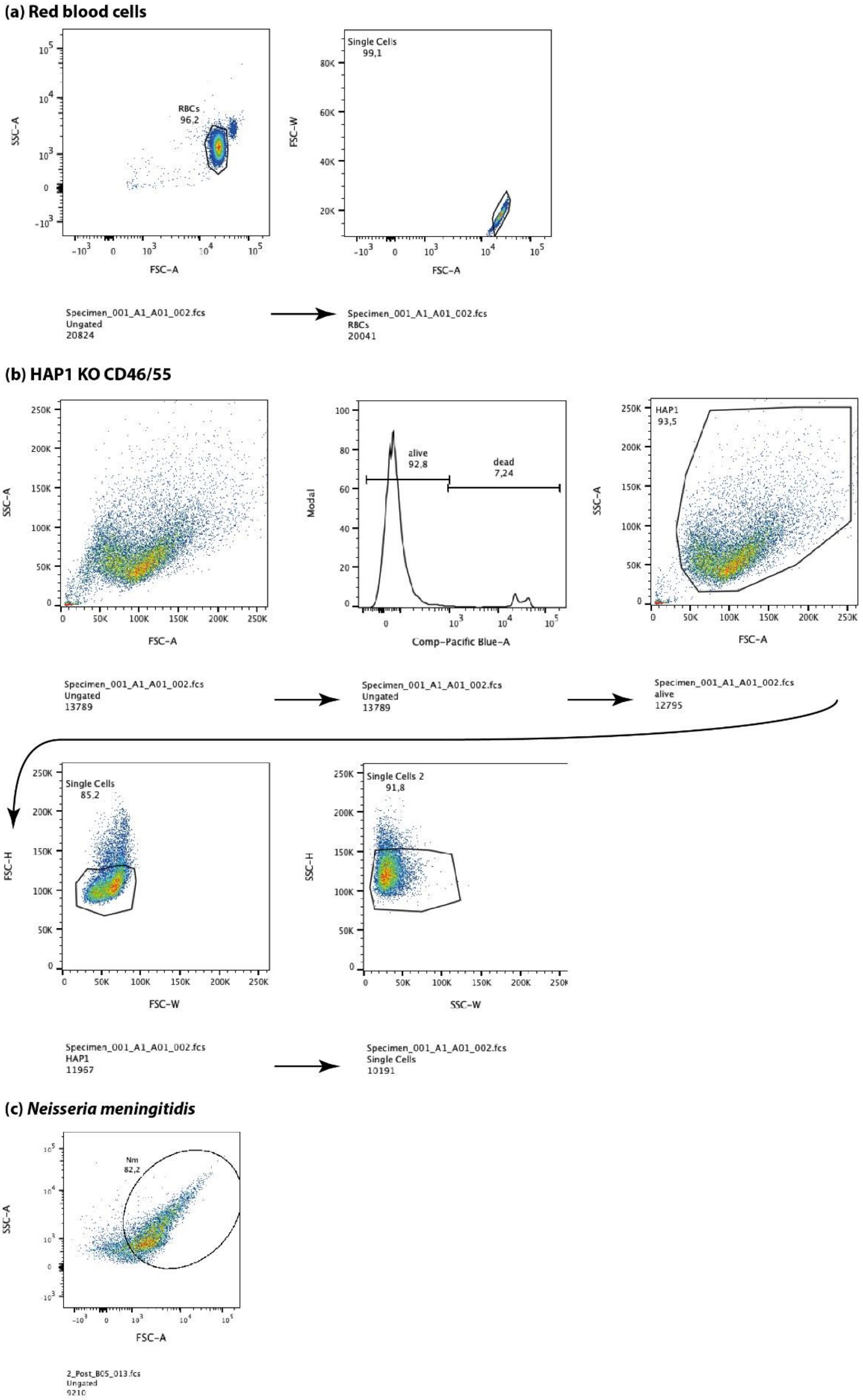

Supplementary figure 1. FACS gating strategies. (a) Cells were gated on red blood cells using SSC-A vs. FSC-A and then on single cells using FSC-W vs. FSC-A. **(b)** HAP1 CD56/CD55 KO cells were stained with DAPI to gate on living cells, then on SSC-A vs. FSC-A, FSC-H vs. FSC-W and SSC-H vs. SSC-W for single HAP1 cells. **(c)** Cells were gated on *N. meningitidis* on SSC-A vs. FSC-A.

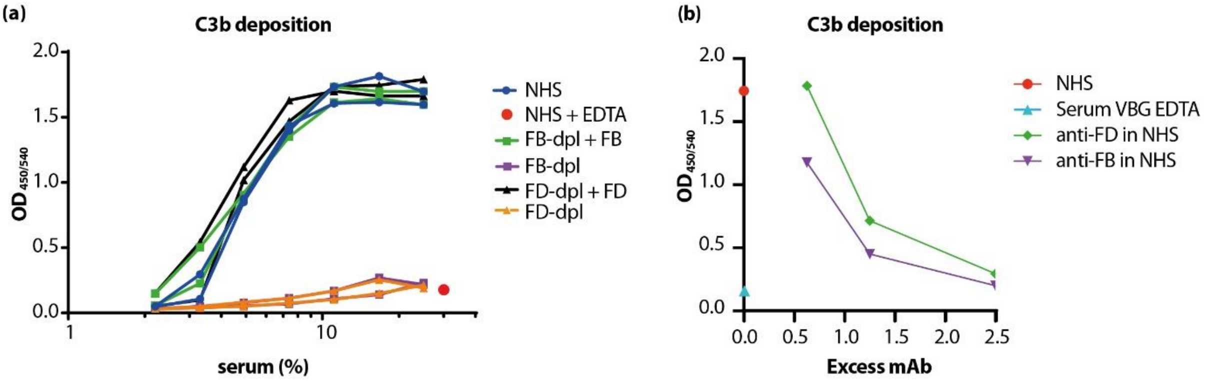

Supplementary figure 2. Sera depleted of and antibodies against FB and FD both fully abrogate AP activation. (a) FB- and FD-depleted sera showed a complete reduction in a C3b deposition ELISA in an AP-specific, Mg-containing buffer, which can be fully restored by the addition of FB and FD respectively. **(b)** The AP-specific C3b deposition ELISA showed that both anti-FB and anti-FD can reduce C3 deposition to the level of serum in EDTA at 2.5-fold excess mAb to serum protein in 20% serum. **a** shows pooled data from two independent experiments (each replicate), **b** for one experiment.

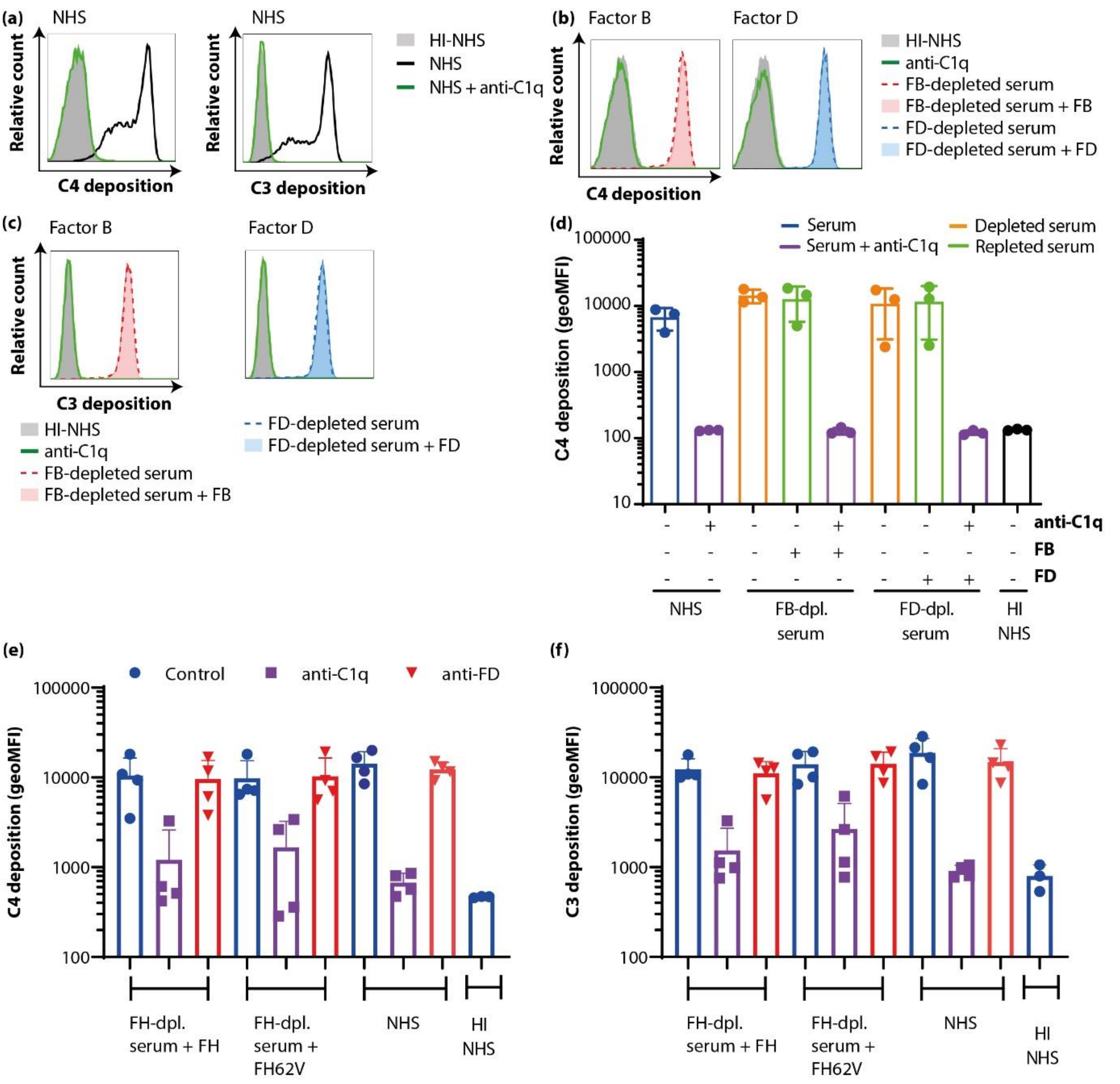

Supplementary figure 3. Complement activation on HAP1 cells lacking complement regulators CD46 and CD55 is not AP dependent. (a) C4 and C3 deposition by NHS on HAP1 ΔCD46/CD55 KO cells is completely inhibited by blocking the CP with anti-C1q. **(b)** C4 and **(c)** C3 deposition occurs on HAP1 CD46/CD55 KO cells both with and without FB and FD. **(d)** Quantification of the C4 deposition on HAP1 CD46/CD55 KO cells in NHS, FB- and FD-depleted serum with/without anti-C1q, FB and FD. **(e, f)** Quantification of C4 (**e**) and C3 (**f**) deposition on HAP1 CD46/CD55 KO cells by FH-depleted serum reconsituted with FH or FH$_{62V,}$ NHS or HI-NHS, with/without anti-C1q or anti-FD. Inhibition of the AP or CP both shows a similar result between wildtype FH and FH$_{62V.}$ **a–c** are representative graphs of n = 3, **d** shows mean + SD of n = 3, **e** and **f** show mean + SD of n = 4. Significance was tested using Friedman test with Dunn's multiple comparisons test for **d** and with Kruskal-Wallis test with Dunn's multiple comparisons test for **e** and **f**.

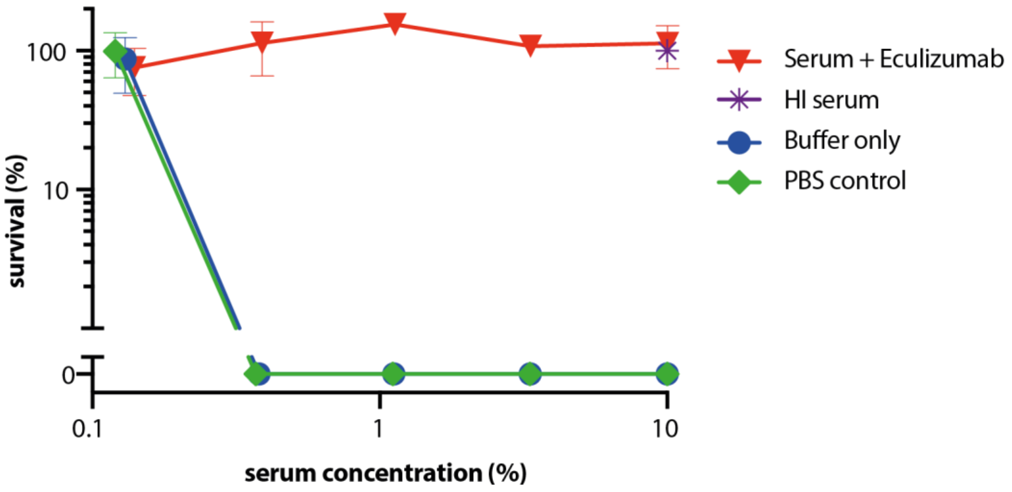

Supplementary figure 4. Bacterial killing of *E. coli* by serum is complement-mediated bacterial killing by serum is completely inhibited by eculizumab and HI NHS, indicating that this is mediated by the complement system. The f igure shows the mean with SD from n = 3.
